# Structure and Function of p53-DNA Complexes with Inactivation and Rescue Mutations: A Molecular Dynamics Simulation Study

**DOI:** 10.1371/journal.pone.0134638

**Published:** 2015-08-05

**Authors:** Balu Kamaraj, Annemie Bogaerts

**Affiliations:** Research group PLASMANT, Department of Chemistry, University of Antwerp, Universiteitsplein 1, 2610, Wilrijk-Antwerp, Belgium; University of Akron, UNITED STATES

## Abstract

The tumor suppressor protein p53 can lose its function upon DNA-contact mutations (R273C and R273H) in the core DNA-binding domain. The activity can be restored by second-site suppressor or rescue mutations (R273C_T284R, R273H_T284R, and R273H_S240R). In this paper, we elucidate the structural and functional consequence of p53 proteins upon DNA-contact mutations and rescue mutations and the underlying mechanisms at the atomic level by means of molecular dynamics simulations. Furthermore, we also apply the docking approach to investigate the binding phenomena between the p53 protein and DNA upon DNA-contact mutations and rescue mutations. This study clearly illustrates that, due to DNA-contact mutants, the p53 structure loses its stability and becomes more rigid than the native protein. This structural loss might affect the p53-DNA interaction and leads to inhibition of the cancer suppression. Rescue mutants (R273C_T284R, R273H_T284R and R273H_S240R) can restore the functional activity of the p53 protein upon DNA-contact mutations and show a good interaction between the p53 protein and a DNA molecule, which may lead to reactivate the cancer suppression function. Understanding the effects of p53 cancer and rescue mutations at the molecular level will be helpful for designing drugs for p53 associated cancer diseases. These drugs should be designed so that they can help to inhibit the abnormal function of the p53 protein and to reactivate the p53 function (cell apoptosis) to treat human cancer.

## Introduction

In this paper, we aim to observe the structural and functional behavior of p53 proteins upon DNA-contact mutations (R273H and R273C) and rescue or second suppressor mutations (T284R/S240R). p53 is a tumor suppressor protein which is encoded by the P53 gene. P53 is also called “the guardian of the genome” and it plays an essential role in cell cycle regulation, like cell apoptosis. P53 is mainly involved in control and monitoring the cell division [1–4]. The P53 gene encodes a protein called p53, which is a homo-tetramer, consisting of 393 amino acids [5–7]. The N-terminal region (residues 1–62) contains the transactivation domain, which is further divided into two subdomains, and it is followed by a proline-rich region (residues 63–64), important for apoptotic activity [8, 9]. The DNA-binding domain (DBD), also known as the p53 core domain (p53C) (residues 94–292), contains several electropositive arginine amino acids and one zinc atom, which interact with the DNA (5'-D(*CP*GP*GP*GP*CP*AP*TP*GP*CP*CP*CP*G)-3') molecule [10]. The nuclear localization signaling domain (residues 316–325) is involved in the intracellular localization of p53. The oligomerization domain (OD) (residues 326–356) is responsible for tetramerization, which is essential for the p53 activity. Finally, the C-terminal regulatory domain (CTD) (residues 363–393) acts as a flexible region, and is involved in the down-regulation of the central DNA binding domain [11–13].

The most frequently mutated region in p53 in human cancer is the DBD. Most of the mutations are missense mutations present frequently in the DBD, leading to loss of target gene transactivation [14]. The functional effect of p53 is linked to DNA damage. It is clearly proven that p53 plays a crucial role in cancer progression [15–22] as well as in different physiological [23] and anti-cancer responses [24]. The following six residues of p53, i.e., Arg248, Arg273, Arg175, Gly245, Arg249 and Arg282, are frequently mutated in human cancer [25]. Most of the tumor-related p53 mutations, called hotspot mutations, occur in the DNA-binding core domain of p53. Arg273(R273), a DNA-contact amino acid, is one of the most frequently altered amino acid residues in human cancer, and it mutates into histidine (46.6%) and to cysteine (39.1%) [26, 27].

**Fig 1:**
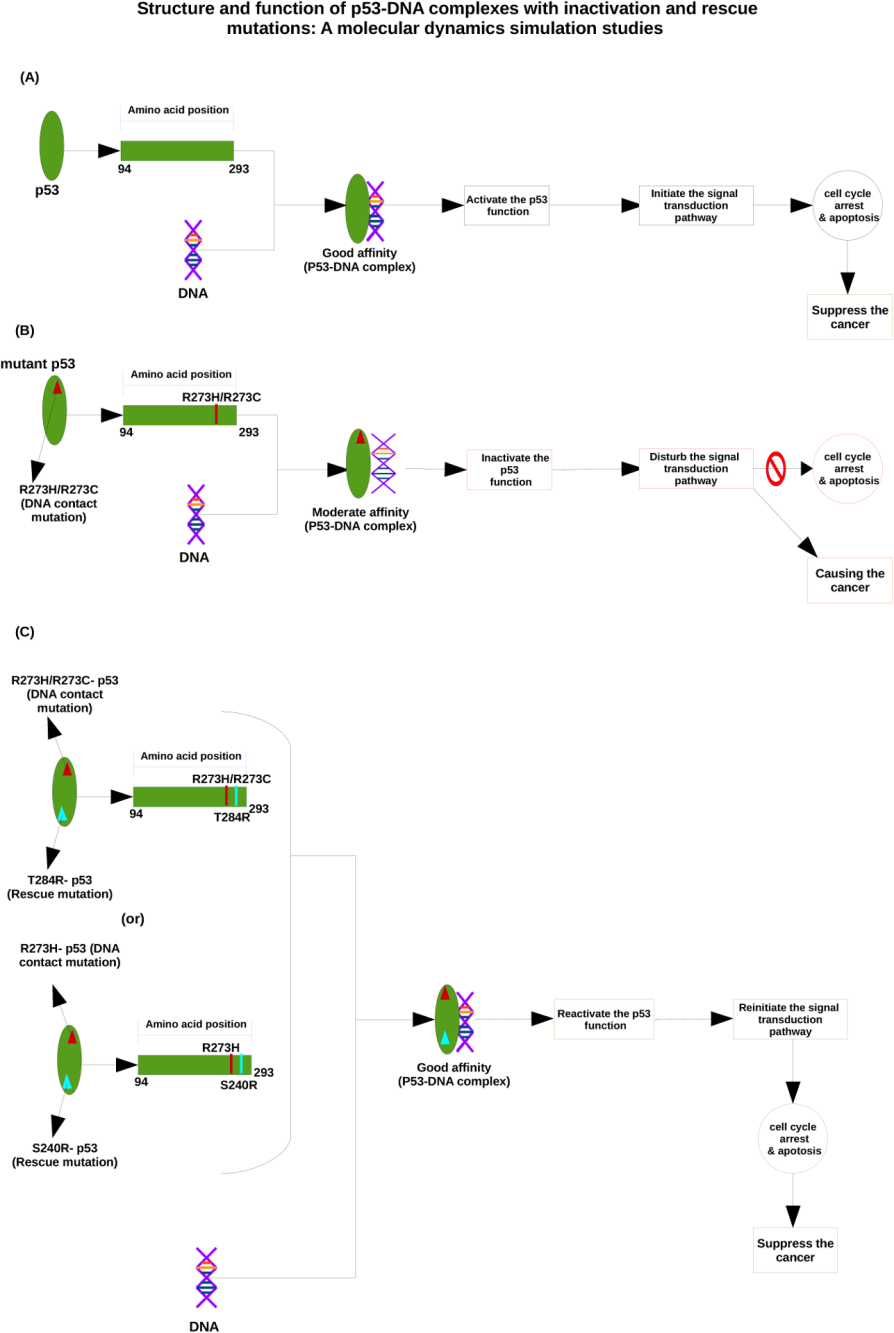
Mechanism of p53-DNA interaction upon DNA-contact and rescue mutations. (A) Shows the normal p53-DNA interaction, leading to cancer suppression. (B) Illustrates how a DNA-contact mutation results in a reduced p53-DNA affinity, which is a possible cause of cancer. (C) Shows that a rescue mutation can restore the good p53-DNA affinity, and thus give again rise to cancer suppression.

The PDB structures of the p53 coredomain bound to DNA [28–34] show that the guanidinium groups of the R273 residues (positively charged) interact with the DNA backbone (negatively charged) at the center of each DNA half-site, which is supported by hydrogen bonding and salt-bridge interactions. TheR273 residues play an important role in docking, to study the p53 interaction with the DNA backbone [35, 36]. Substitution of R273 by histidine or cysteine amino acid residues, referred to as R273H and R273C, leads to a dramatic reduction in the DNA binding affinity [37]. Inactivation of the resulting mutant p53 function is a tough challenge. Reversing the effect of single mutations (R273H and R273C) in the p53 core domain is rescued by second-site suppressor mutations (T284R, S240R), which leads to a reactivation of the normal p53 activity (i.e., DNA binding, transcriptional activation and tumor-suppressing activity)[34]. Mutant T284R (i.e., substitution of threonine by arginine at position 284) could restore the activity of R273H and R273C mutations [38] whereas mutant S240R (i.e., substitution of serine by arginine at position 240), could rescue the R273H mutation [39]. The overall mechanism of native, DNA-contact mutants and rescue mutants of the p53-DNA complex is shown in Fig 1.

The main objective of this paper is to elucidate the structural and functional behavior of p53 proteins upon DNA-contact mutations (R273H and R273C) and rescue or second suppressor mutations (T284R/S240R) and the corresponding mechanism at the atomic level, by means of molecular dynamics simulations. Furthermore, we also investigate the p53-DNA interaction behavior upon DNA contact mutants(R273H and R273C) and rescue mutants(T284R/S240R) by means of the docking approach. In this study we observe that DNA contact mutants (R273H and R273C) get damaged and become more rigid in nature, and this structural rigidity affects the p53-DNA interaction pattern and it results in loss of its activity. On the other hand, the rescue or second suppressor mutants (R273H_T284R, R273C_T284R and R273H_S240R) behave like the native protein, so they rescue the normal activity of the p53 protein and restore the favorable interaction with the DNA molecule. Therefore, it might reactivate the function of p53, which initiates cell cycle arrest and apoptosis (the signal transduction pathway) in the cell and suppresses human cancer.

## Materials and Methods

### Datasets

The crystal structure of native (PDB ID: 2AC0_A), DNA-contact mutants R273C (PDB ID: 4IBQ_A) and R273H (PDB ID: 4IBS_A) and rescue mutants R273C_T284R (PDB ID: 4IBZ_A), R273H_T284R (PDB ID: 4IBT_A) and R273H_S240R (PDB ID: 4IBY_A) of p53 and DNA (PDB ID: 4IBV_B) [34]. We consider a monomer of native DNA-contact mutants and rescue mutants of p53 proteins (chain A) and a monomer of DNA (chain B) for our studies.

### Molecular dynamics simulations

MD simulations were performed using the GROMACS 4.6.1 package [40]. Native, DNA-contact mutants (R273C and R273H) and rescue mutants (R273C_T284R, R273H_T284R and R273H_S240R) of the p53 structures were used as input structures for the MD simulations. The systems were solvated with TIP3P water molecules in a cubic box with dimensions 1x1x1 nm^3^. Periodic boundary conditions were applied in all three (x, y, z) directions to mimic the infinity of the system. At physiological pH conditions, the structures were found to be positively charged. Hence, we added chloride ions (Cl^-^) to make the system electrically neutral in the simulation box. Initially, the solvent molecules were relaxed, whereas all the solute atoms were harmonically restrained to their original positions with a force constant for 5000 steps. After this, the whole molecular system was subjected to energy minimization for 5000 iterations by the steepest descent algorithm, implementing the Amber ff99SB-ILDN force field. Subsequently, the minimized systems were subjected to MD simulations in two steps [41], i.e., initially under an NVT ensemble (constant number of particles, volume, and temperature) for 1000 ps, followed by an NPT ensemble (constant number of particles, pressure, and temperature) for 1000 ps, each at 300 K with positions restrained for the entire system, except the water molecules, in order to enable a balance of the solvent molecules around the residues of the protein. The Berendsen temperature coupling method was applied to regulate the temperature inside the box. The Particle Mesh Ewald (PME) method [42] was used to treat the long-range electrostatic interactions. The pressure was maintained at 1 atm with an allowed compressibility range of 10^−5^ atm. The LINCS algorithm was used to constrain the bond lengths involving hydrogens, permitting a time step of 2 fs. Van der Waals and Coulomb interactions were truncated at 1.0 nm. The non-bonded pair list was updated every 10 steps and conformations were stored every 0.5 ps. Finally, the systems were subjected to MD simulations for 100 ns each at 300 K without any constraint. The results were analyzed using the in-built analysis package of GROMACS, XMGRACE [43]. We analyzed the root mean square deviations (RMSD), root mean square fluctuations (RMSF), the radius of gyration (Rg), the solvent accessible surface area (SASA) and the number of hydrogen bonds (NH-bonds), and we made a comparison between native, DNA-contact and rescue mutants to examine the structural and functional behaviour of the p53 protein. To further support our MD simulation results, the large scale collective motion of the native, DNA-contact and rescue mutants of p53 were studied by means of essential dynamics (ED) analysis [44]. The dynamics of two proteins were best characterized through their phase space behaviour. The eigenvectors of the covariance matrix were called its principal components. The change of a particular trajectory along each eigenvector was obtained by this projection.

### Root mean square deviation (RMSD) cut-offcluster

In order to select a reduced set of representative models of the native, DNA-contact and rescue mutant p53 protein, RMSD conformational clustering was performed using the Gromos method [45] implemented in GROMACS (g_cluster). In the Gromos clustering algorithm, the conformation with the highest number of neighbors, identified within the chosen RMSD cut-off, is chosen as the center of the first cluster. All the neighbors of this conformation are removed from the ensemble of conformations. The center of the second cluster is then determined in the same way, and the procedure is repeated until each structure has been assigned to a cluster [45].

### Protein-DNA interaction analysis

The HADDOCK protocol [46, 47] was used to dock the native, DNA-contact mutants (R273C and R273H) and rescue mutants (R273C_T284R, R273H_T284R and R273H_S240R) of the p53 protein with the DNA molecule. HADDOCK is a series of scripts that run in combination with ARIA [48, 49] and CNS [50]. The docking process in HADDOCK is driven by ambiguous interaction restraints (AIRs), which are derived from the available experimental information on the residues involved in the intermolecular interaction. The predicted interface surface residues in p53 (position number 94, 95, 96, 97, 98, 112, 136, 137, 138, 144, 146, 172, 174, 176, 177, 178, 179, 180, 181, 182, 183, 184, 185, 186, 187, 188, 190, 192, 197, 198, 199, 200, 201, 202, 204, 205, 206, 210, 212, 213, 214, 219, 233, 237, 239, 240, 241, 242, 243, 244, 245, 247, 248, 264, 275 and 276) together with interface residues on the partner DNA molecule (residues between 1 and 12) were used as active residues, while the residues neighbouring the active residues were used as passive residues. At the first stage of the docking protocol, which consists of randomization of the orientations and rigid body energy minimization, we calculated 1,000 complex structures. The 200 solutions with the lowest intermolecular energies were selected for semi-flexible simulated annealing in torsion angle space. The resulting structures were then refined in explicit water. Finally, the solutions were clustered using a threshold value of 1.5 Å for the pairwise backbone root mean square deviation (RMSD) at the interface, and the resulting clusters were ranked according to their average interaction energy (defined as the sum of van der Waals, electrostatic and AIRs energy terms) and their buried surface area. One lowest energy structure of the lowest intermolecular energy cluster was selected for analysis. This lowest energy structure displayed no AIR restraint violations (within a threshold of 0.3 Å) and was accepted as the final docked structure for the complex.

HADDOCK scoring was performed according to the weighted sum (HADDOCK score) of different energy terms, which include van der Waals energy, electrostatic energy, distance restraints energy, inter-vector projection angle restraints energy, diffusion anisotropy energy, dihedral angle restraints energy, symmetry restraints energy, binding energy, desolvation energy and buried surface area. Along with the HADDOCK score, we calculated the van der Waals energy, electrostatic energy, restraints violation energy, desolvation energy and buried surface area. Intermolecular contacts (hydrogen bonds and non-bonded contacts) were analysed with the LIGPLOT software [51]. The default settings were used (3.9 Å heavy atoms distance cut-off for non-bonded contacts; 2.7 and 3.5 Å proton–acceptor and donor–acceptor distance cut-offs, respectively, with minimum 90° angles (D–H–A, H–A–AA, D–A–AA) for hydrogen bonds) [53].

## Results and Discussion

### Structural and functional analysis of the p53 protein upon DNA-contact mutations and rescue mutations

**Fig 2:**
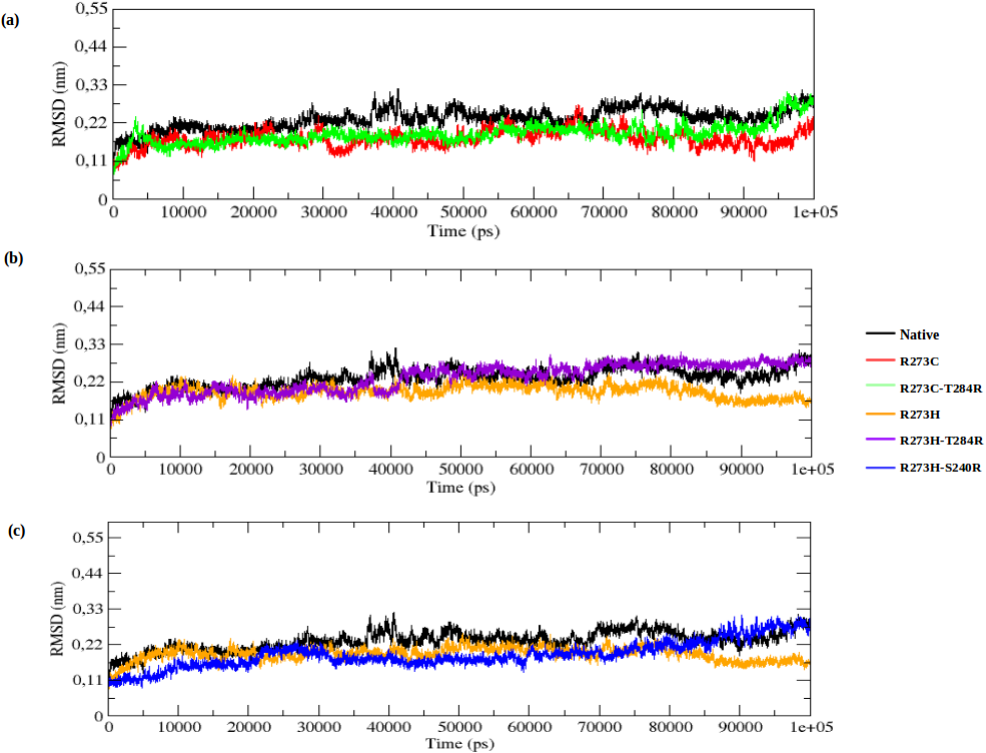
RMSD of native, DNA-contact (R273C and R273H) and rescue mutants (R273C_T284R, R273H_T284R and R273H_S240R) of the p53 protein versus time at 300K. (a) Native, R273C and R273C_T284R, (b) Native, R273H and R273H_T284R, (c) Native, R273H and R273H_S240R.

The MD simulations allow us to understand the structural and functional behaviour of the p53 protein upon DNA-contact (R273C and R273H) and their rescue mutations (R273C_T284R, R273H_T284R and R273H_S240R). We studied the RMSD, RMSF, Rg, SASA, and the number of hydrogen bonds (NH-bonds). We also performed ED analysis and cluster analysis, and made a comparison between the native and DNA-contact (R273C and R273H) and rescue mutations (R273C_T284R, R273H_T284R and R273H_S240R) of the p53 protein. The RMSD for all C_α_-atoms from the starting structure was analyzed to study the convergence of the protein system. In the RMSD plot, the native and R273C mutant structure show a similar way of deviation from the start till ~ 8000 ps, after which the mutant (R273C) structure shows a decrease in RMSD value in comparison to the native structure till the end of the simulation. The rescue mutation (T284R) of the R273C mutant structure shows a similar way of deviation as the native structure from the start to ~ 6500 ps, but then it shows the same way of deviation as the DNA-contact mutant (R273C), till 85,000ps, after which it rises and becomes similar to the native structure at the timescale of ~95,000 ps, as illustrated in Fig 2A. The average RMSD value of the native, DNA-contact mutant R273C and its rescue mutant (R273C_T284R) are presented in S1 Table. In Fig 2B and 2C, the native and mutant R273H show a similar way of deviation from the start till ~21,000 ps. Subsequently, the mutant (R273H) structure shows a smaller deviation till the end of the simulations, while the rescue mutation (T284R) of the R273H mutant and the native structure show the same extent of deviation from the start till ~27,000 ps and again from ~ 41,000 ps to the end of the simulations. Another rescue mutation (S240R) of the R273H mutant structure shows less deviation than the native structure from the start to ~83,000 ps, but afterwards, it shows the same deviations to the end of the simulation (see Fig 2C). The average RMSD values of the DNA-contact mutant R273H and its rescue mutants (R273H_T284R and R273H_S240R) are also presented in S1 Table. This Table indicates that, due to the DNA-contact mutants (R273C and R273H), p53 loses its stability and this affects the structural orientation of the p53 protein, whereas the rescue mutants(R273C_T284R, R273H_T284R and R273H_S240R) restore to some extent the stability of the p53 protein upon DNA-contact mutation (R273C and R273H). This, in turn, leads to reactivate the function of the p53 protein.

**Fig 3:**
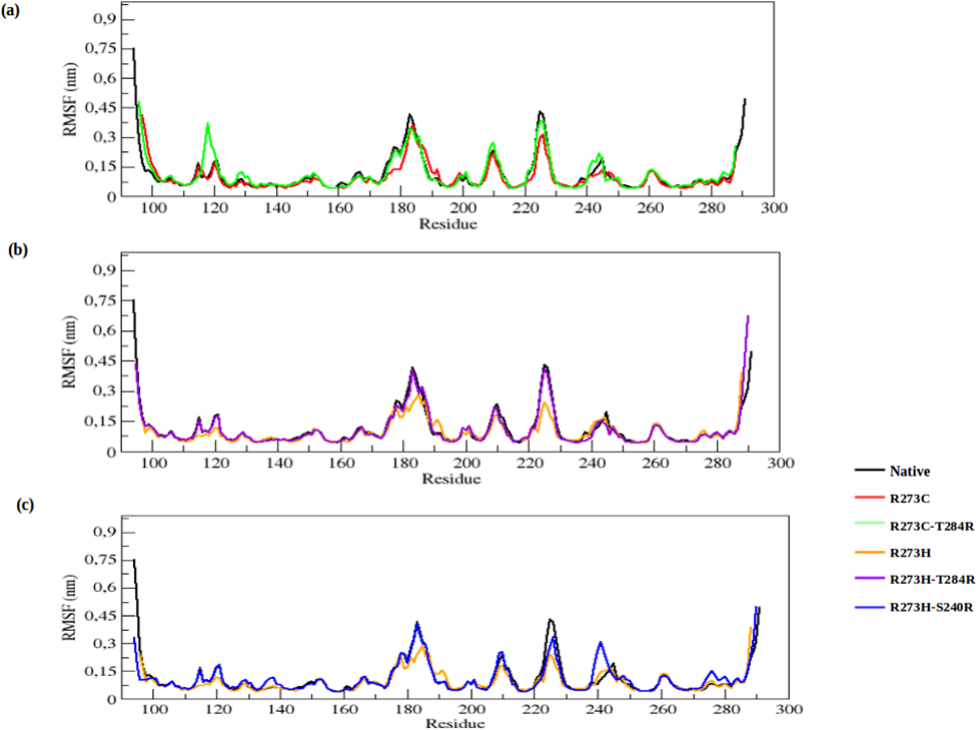
RMSF of the backbone of C-alpha atoms of native, DNA-contact (R273C and R273H) and rescue mutants (R273C_T284R, R273H_T284R and R273H_S240R) of the p53 protein versus residue at 300K. (a) Native, R273C and R273C_T284R, (b) Native, R273H and R273H_T284R, (c) Native, R273H and R273H_S240R.

To determine the dynamic behaviour of the p53 protein residues upon DNA-contact (R273C and R273H) and rescue mutations (R273C_T284R, R273H_T284R and R273H_S240R), the RMSF of native, DNA-contact and rescue mutant structures are depicted in Fig 3A–3C. The DNA-contact mutations (R273C and R273H) show a lower degree of flexibility than native p53, whereas the rescue mutations (R273C_T284R, R273H_T284R and R273H_S240R) show a similar flexibility as native p53 throughout the simulation time. This further illustrates that the DNA-contact mutants lose the flexible conformation and become more rigid whereas the rescue mutants can restore the flexible conformation in the p53 protein.

**Fig 4:**
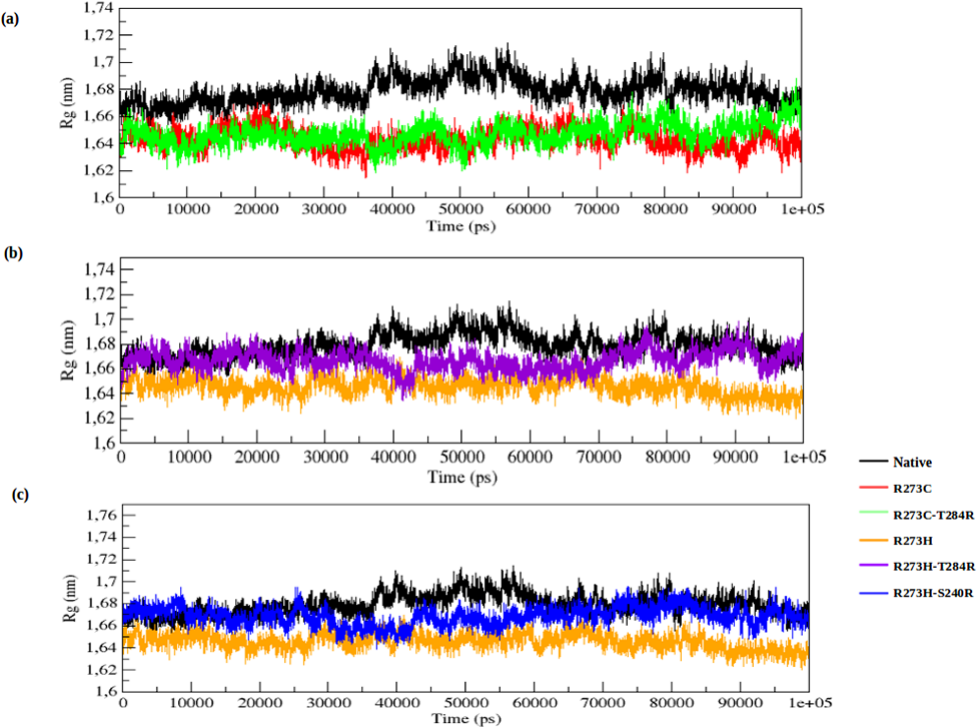
Radius of gyration of C-alpha atoms of native, DNA-contact (R273C and R273H) and rescue mutants (R273C_T284R, R273H_T284R and R273H_S240R) of the p53 protein versus time at 300K. (a) Native, R273C and R273C_T284R, (b) Native, R273H and R273H_T284R, (c) Native, R273H and R273H_S240R.

The Rg parameter provides an indicative level of compaction in the protein structure. It is defined as the mass-weighted root mean square distance of the collection of atoms from their common center of mass. In the Rg plot (Fig 4A), the DNA-contact mutant (R273C) shows a lower Rg value than the native structure from the start to the end of the simulation. The rescue mutant (R273C_T284R) shows a similar Rg value as the DNA-contact mutant (R273C) from the start to ~95,500 ps, after which it rises and reaches the Rg value of the native structure. The average Rg values of the native, DNA-contact (R273C) and rescue mutant (R273C_T284R) are again listed in S1 Table. Fig 4B and 4C show more convincingly that the DNA-contact mutant (R273H) has a lower Rg value than the native structure, whereas both rescue mutants (R273H_T284R and R273H_S240R) show almost the same Rg value as the native structure from the start to ~30,500 ps and again from ~70,000 ps to the end of the simulation. The average Rg values of the contact (R273H) and rescue mutations (R273H_T284R and R273H_S240R) are also presented in S1 Table.

**Fig 5:**
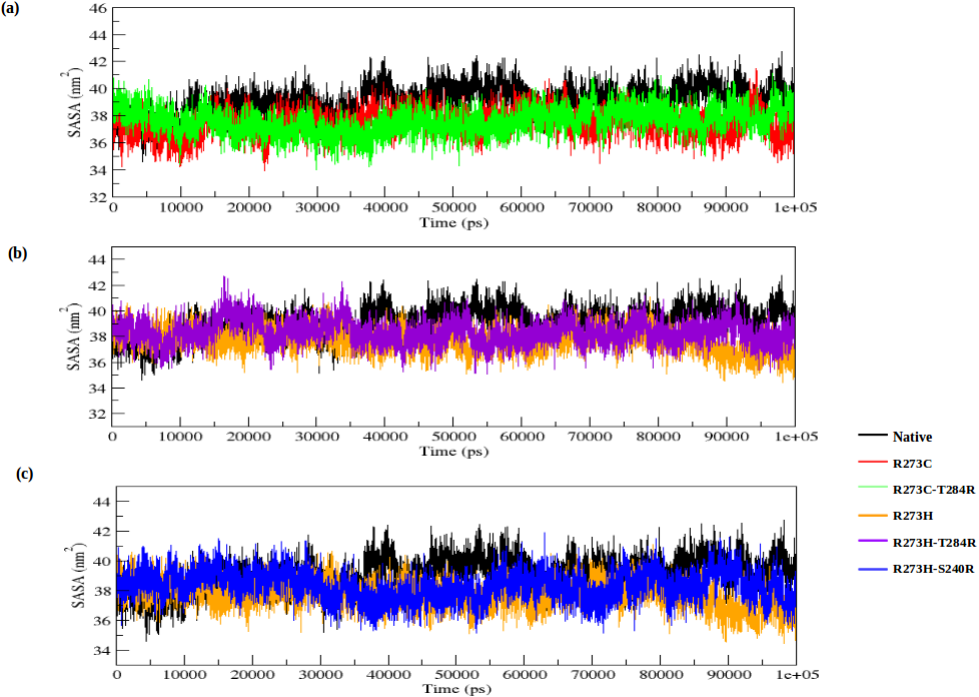
Solvent accessible surface area (SASA) of native, DNA-contact (R273C and R273H) and rescue mutants (R273C_T284R, R273H_T284R and R273H_S240R) of the p53 protein versus time at 300K. (a) Native, R273C and R273C_T284R, (b) Native, R273H and R273H_T284R, (c) Native, R273H and R273H_S240R.

Fig 5 illustrates the change of SASA for the native, DNA-contact (R273C and R273H) and rescue mutants (R273C_T284R, R273H_T284R and R273H_S240R) of the p53 protein with time. SASA is the surface area of the biomolecules that is accessible to the solvent. The average SASA values of the native, DNA-contact and rescue mutants are again listed in S1 Table. The lower fluctuation in Rg plots in both DNA-contact mutant structures indicates that the protein might be undergoing a significant structural transition. This is further supported by the SASA result, where the DNA-contact mutants (R273C and R273H) exhibit lower values of SASA as compared to the native p53 protein. A lower value of SASA in the DNA-contact mutant structures denotes its relatively shrunken nature as compared to the native p53 structure. The rescue mutants, on the other hand, can reach the fluctuation of the native p53 protein at the end of the simulation in both the Rg and SASA plot (Fig 4A–4C and Fig 5A–5C). It indicates that the rescue mutants (R273C_T284R, R273H_T284R and R273H_S240R) can restore the structural changes in the p53 protein upon DNA-contact mutant (R273C and R273H) structures.

**Fig 6:**
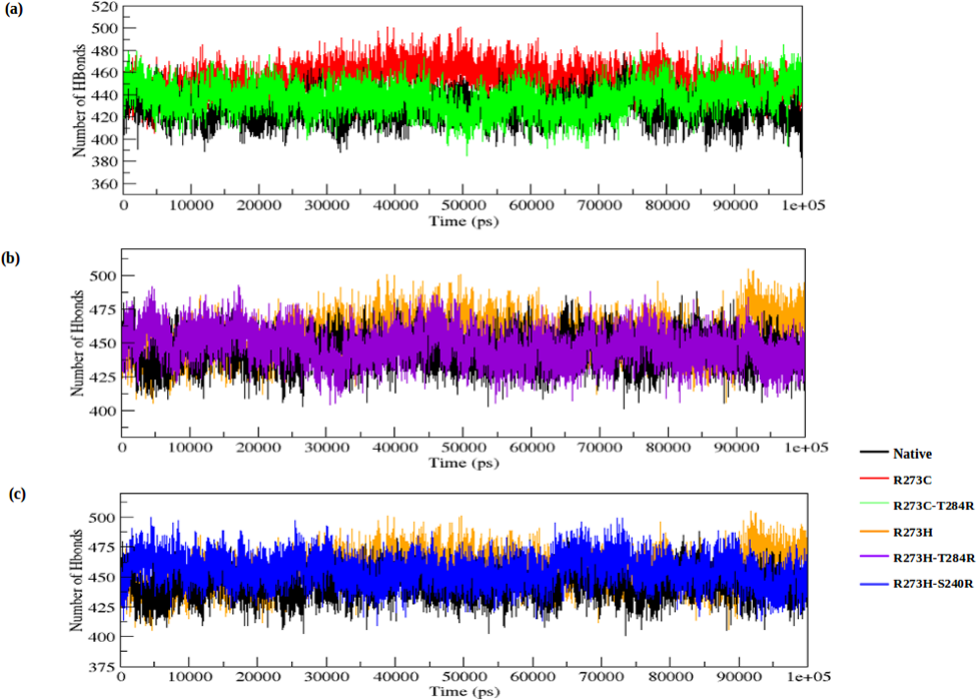
Average number of intermolecular hydrogen bonds in native, DNA-contact (R273C and R273H) and rescue mutants (R273C_T284R, R273H_T284R and R273H_S240R) of the p53 protein versus time at 300K. (a) Native, R273C and R273C_T284R, (b) Native, R273H and R273H_T284R, (c) Native, R273H and R273H_S240R.

We also observed notable differences in the NH-bond pattern during the simulation. The hydrogen bonds account for a major factor of maintaining the stable conformation of the protein. NH-bond analysis of native and DNA-contact and rescue mutations of the p53 proteins was performed with respect to time in order to understand the relationship between flexibility and hydrogen bond formation. The DNA-contact mutants (R273C and R273H) show a slightly larger number of NH-bonds formed during the simulation than the native p53 protein (see Fig 6A–6C). The rescue mutants (R273C_T284R, R273H_T284R and R273H_S240R) show less NH-bonds than the DNA-contact mutants and show almost a similar number of H-bonds as the native p53 protein. The average number of NH-bonds is given in S1 Table. The NH bond results of the native, DNA-contact and rescue mutant structures correlate well with the RMSD, RMSF, Rg and SASA plot results. The results indicate that the p53 protein conformation becomes more rigid in nature upon DNA-contact mutants (R273C and R273H), which affects the functional behaviour of the p53 protein. On the other hand, the second site suppressor or rescue mutants (R273C_T284R, R273H_T284R and R273H_S240R) alter the structural disturbance of the p53 protein upon DNA-contact mutants, and can thus rescue the structural and functional behaviour of the p53 protein.

**Fig 7:**
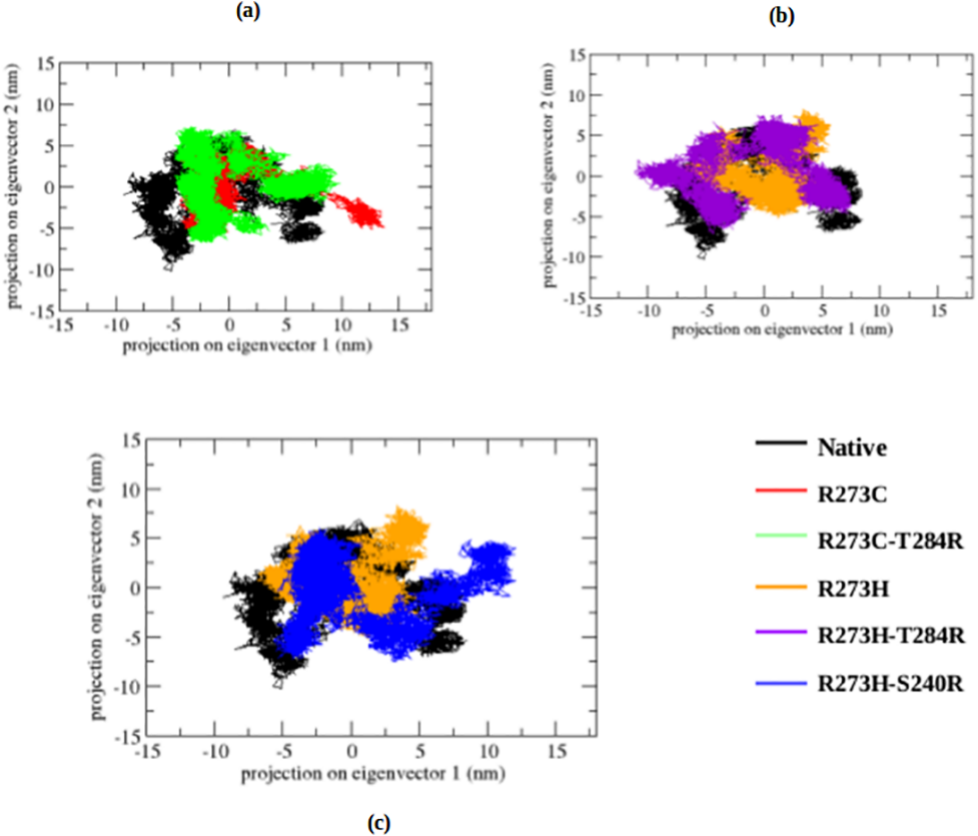
Projection of the motion of the p53 protein in phase space along the first two principal eigenvectors at 300 K. (a) Native, R273C and R273C_T284R, (b) Native, R273H and R273H_T284R, (c) Native, R273H and R273H_S240R.

To further support our results, we performed an essential dynamics (ED) analysis to obtain a better view of the dynamical mechanical property of the investigated structures. In ED analysis, we applied a simple linear transformation in the Cartesian coordinate space and the diagonalization of the covariance matrix. It yields a set of eigenvectors, which gives a vectorial depiction of every single component of the motion indicative of the direction of motion. Within the top eigenvectors, the first two accounted for a significant amount of overall motion in each case. Each eigenvector has a corresponding eigenvalue, which describes the energetic involvement of each component to the motion. The projection of trajectories obtained at 300 K onto the first two principal components (PC1, PC2) shows the motion of native and DNA-contact and rescue mutant p53 proteins in phase space and is illustrated in Fig 7A–7C. In the ED analysis, both DNA-contact mutants (R273C and R273H) cover a smaller region of phase space than the native structure, particularly along the PC2 and PC1 plane, whereas the rescue mutations of R273C (R273C_T284R) and R273H (R273H_T284R and R273H_S240R) show a similar phase space behaviour as the native protein. The overall flexibility of the native structure, and the DNA-contact and rescue mutants was calculated by the trace of the diagonalized covariance matrix of the C_α_-atomic positional fluctuations (S1 Table: covariance value). From Fig 7A–7C and S1 Table, it is clear that the overall flexibility was reduced in both DNA-contact (R273C and R273H) mutants compared to the native structure at 300K. Moreover, the rescue mutants can restore the flexibility in the p53 protein upon DNA-contact mutants and show a similar way of motions as the native protein, thus restoring the normal function of the p53 protein upon DNA-contact mutations.

Finally, we also performed cluster analysis to investigate the effect of the DNA-contact and rescue mutations on the structural and functional behaviour of the p53 protein. Clustering is one way of analyzing the data from an extensive sampling of a high-dimensional phase space, such as that obtained from MD calculations. We clustered the sampled configurations in each trajectory based on the method by Daura et al [45], to report the conformation flexibility of the p53 protein upon DNA-contact and rescue mutations. We ranked each trajectory frame based on the number of neighboring frames that were within 1 Å RMSD. The top-ranked frame, together with all its neighbors, was then removed and the ranking process was repeated.

Representative structures for a member of one of the most populated clusters of the native structure, the DNA-contact mutant (R273C and R273H) and rescue mutant (R273C_T284R, R273H_T284R and R273H_S240R) structures are shown in S1 Fig. The statistics of the cluster analysis are listed in S2 Table. The significant number of clusters obtained for the native p53 protein illustrates its flexibility. The DNA-contact mutant (R273C and R273H) structures show a lower number of clusters and more members in the populated clusters than the native protein, whereas the rescue mutants show again a larger number of clusters and less members in the populated clusters than the DNA-contact mutant structures. The analysis clearly shows that the members of the native structure are more flexible and exhibit more structural deviation than the DNA-contact mutant (R273C and R273H) structures, which have a more rigid conformation and shows less deviation from each other. This observation clearly indicates again that the p53 protein loses its flexible conformation due to DNA-contact mutation and becomes more rigid in nature, whereas the rescue mutants can restore the flexible conformation of the native structure, which may lead to reactivate the function of the p53 protein.

### Protein-DNA interactions

The MD results clearly imply that the DNA-contact mutation of the p53 protein affects its flexibility, and thus its function (i.e., DNA-p53 protein interaction) and this may lead to cancer. The rescue mutations, however, reach again the native conformation at the end of the simulation, and this may reactivate the function of the p53 protein and suppress the cancer activity. To verify this further, we applied a molecular docking method to evaluate the interaction between the p53 protein and a DNA molecule upon DNA-contact mutations and rescue mutations.

HADDOCK is a method that directly allows the incorporation of biological and/or biophysical information (protein-protein, protein-DNA and protein-RNA) to drive the docking. In this study we docked the MD output of the native, DNA-contact and rescue mutants of the p53 protein (monomer) with a DNA (monomer) molecule to observe the interaction or the affinity loss between the biomolecules upon mutation. Calculation of the HADDOCK score is essential to understand the affinity level between the biological partners. A HADDOCK score is determined for each structure after docking, allowing to rank the structures. The score is a weighted sum of the intermolecular electrostatic (Elec), van der Waals (vdw), desolvation (Dsolv), AIR energies and a buried surface area (BSA) [52–54].

The native p53-DNA complex shows a HADDOCK score of -92.8 ± 2.6, while the DNA-contact mutants (R273C-DNA and R273H-DNA) of the p53-DNA complexes show a score of -74.2 ± 10.5 and -73.7 ± 5.9, respectively (S3 Table), and the score of the rescue mutants (R273C_T284R, R273H_T284R, R273H_S240R) of the p53-DNA complexes amount to -85.6 ± 5.7, -83.9 ± 18.2 and -84.3 ± 1.4, respectively. The less negative values of the HADDOCK score of the DNA-contact mutant (R273C and R273H) complexes indicate a lower affinity between the biological partners (p53 protein-DNA) compared to the native complex, while the rescue mutants of the p53-DNA complexes have again a similar HADDOCK score as the native p53 protein-DNA complex, illustrating again a good affinity between the biological partners.

The buried surface area (BSA) is used to quantify the protein surface which is not exposed to water. The native p53-DNA complex shows a BSA value of 1994.7 ± 87.1, while the BSA values of the DNA-contact mutants (R273C-DNA and R273H-DNA) of the p53-DNA complexes are 1823.3 ± 72.6 and 1845.6 ± 132.1, respectively, and the rescue mutants (R273C_T284R, R273H_T284R, R273H_S240R) of the p53-DNA complexes exhibit a BSA score of 2091.2 ± 65.5, 2085.8 ± 68.2 and 1925.7 ± 111.4, respectively (see S3 Table). A higher BSA value enables a close proximity between the biomolecules. The desolvation energy, the restraints violation energy and the BSA have a good correlation with the docking score of the complex during docking. From S3 Table, it is thus again clear that the p53 protein loses its interaction with the DNA molecule due to the DNA-contact mutants (R273C and R273H), whereas the rescue mutants (R273C_T284R, R273H_T284R and R273H_S240R) in the p53 protein can restore the interaction within the p53-DNA complex.

Hydrogen bonds are by far the most important specific interactions in biological recognition processes and are particularly essential in determining the binding specificity [55–60]. The intermolecular hydrogen bonds can provide favorable binding energy [61, 62]. p53 transcription factors are also involved in hydrogen bonding between the protein side chains and the major groove edges of the base pairs (base and shape readout modes) [63, 64]. Generally, bidentate hydrogen bonds between the arginine side chains and guanine bases contribute to a highly specific sequence readout. In a similar way, both transcription factors use shape readout of minor groove geometry and electrostatic potential to enhance the binding specificity. DNA targets of p53 alter their shape through a transition of some base pairs from Watson–Crick to Hoogsteen geometry [36]. Based on this principle, the DNA-contact mutants (R273C and R273H) have lost their binding affinity between the p53 protein and the DNA molecule and this affects their function. The rescue mutants (R273C_T284R, R273H_T284R and R273H_S240R) can retain their binding affinity with DNA, like the native complex (S4 Table).

**Fig 8:**
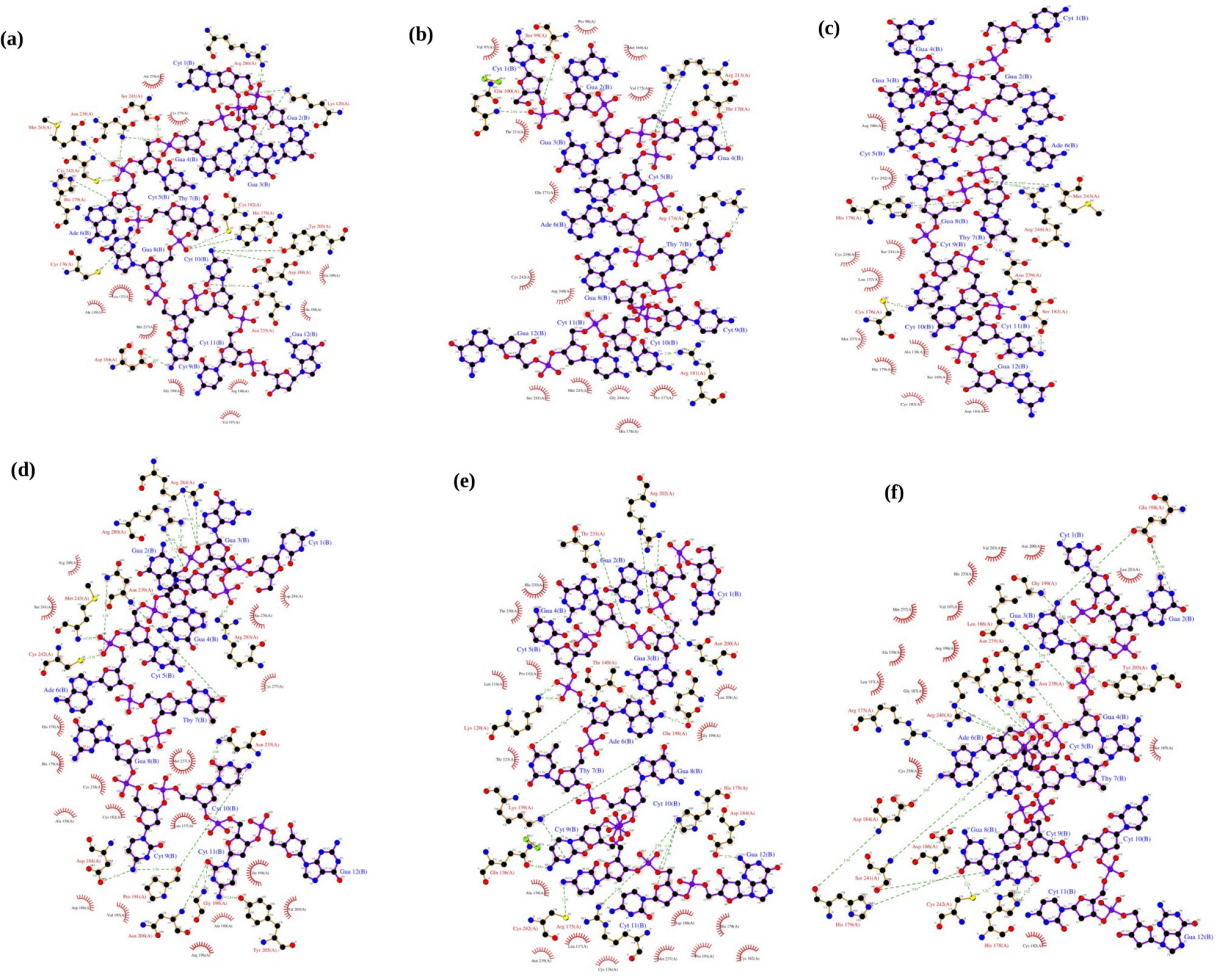
Residue interaction at the protein-DNA interface in the p53-DNA complex. (a) Native-DNA complex, (b) R273C-DNA complex, (c) R273H-DNA complex, (d) R273C_T284R-DNA complex, (e) R273H_T284R-DNA complex and (f) R273H_S240R-DNA complex. The color coding represents the p53 protein in brown color, DNA in purple color. Hydrogen bonding interactions are denoted by dashed lines. This figure was prepared by Ligplot.

The number of intermolecular hydrogen bonds was therefore calculated for the native, DNA-contact mutants (R273C and R273H) and rescue mutants (R273C_T284R, R273H_T284R and R273C-S240R) of the p53-DNA complexes, and the values are listed in S4 Table. The native-DNA complex shows a total of 16 hydrogen bonds between the biomolecules. Arg280, Lys120, Ser241, Asn239, Met243, Cys242, His179, Cys176, Asp184, Asn235, Asp186, Tyr205, Lys182 and His178 act as essential binding site residues in the native-DNA complex (indicated with green dashed lines in Fig 8A). Both DNA-contact mutants (R273C and R273H) of the p53-DNA complexes show only 6 hydrogen bonds (see Fig 8B and 8C). This clearly confirms that the lower number of hydrogen bonds reduces its affinity and also results in the alteration in the binding pattern between the p53 and DNA molecule. The rescue mutants (R273C_T284R, R273H_T284R and R273C-S240R) of the p53-DNA complexes, on the other hand, show 16, 18 and 21 hydrogen bonds, respectively, and they exhibit a similar binding pattern and good affinity with the DNA molecule (Fig 8D–8F).

Thus, the protein-DNA docking analysis and the intermolecular hydrogen bonding patterns confirm that the p53 protein significantly loses its interaction with DNA upon DNA-contact mutations, which can affect the function of the p53 protein and inhibit the cancer suppression, whereas the rescue mutants (R273C_T284R, R273H_T284R and R273H_S240R) in the p53 protein can restore the normal function and may reactivate the cancer suppression function.

## Conclusion

In this study, we investigated the structural and functional behavior of the p53 protein and its binding pattern with a DNA molecule upon DNA-contact (R273C and R273H) and their rescue (R273C_T284R, R273H_T284R and R273H_S240R) mutants. As a result of the DNA-contact mutants (R273C and R273H), the p53 protein loses its stability and becomes more rigid. This might disturb the binding affinity with DNA, which is sufficient to inhibit the suppression of cancer activity. On the other hand, the rescue mutants (R273C_T284R, R273H_T284R and R273H_S240R) can restore the stability loss in the structure and show again a good affinity between the p53 protein and DNA molecules. This could reactivate the function of the p53 protein (cell death) upon DNA-contact mutations (R273C and R273H), and suppress the cancer formation. This insight might help scientists to develop a potential drug target for p53 cancer associated diseases. These drugs should be designed in such a way that they may bind with the p53 protein and lead to inhibition of the abnormal function of p53 and thus to activation of the cell apoptosis mechanism to treat human cancer.

## Supporting Information

S1 Fig Representation of the central conformations representative of the average structure of each cluster of native, DNA-contact (R273C and R273H) and rescue mutants (R273C_T284R, R273H_T284R and R273H_S240R) of the p53 protein. (a) Native, R273C and R273C_T284R, (b) Native, R273H and R273H_T284R, (c) Native, R273H and R273H_S240R.
(TIF)

S1 Table Average values of RMSD, Rg, SASA and number of hydrogen bonds (NH-bonds) of native p53, DNA-contact (R273C and R273H) and rescue mutants (R273C_T284R, R273H_T284R and R27H_S240R). (DOCX)

S2 Table Results of clustering of the backbone of native, DNA-contact (R273C and R273H) and rescue mutant (R273C_T284R, R273H_T284R and R273H_S240R) structures of the p53 protein structural ensemble obtained from the MD trajectories. (DOCX)

S3 Table Statistical analysis of the protein-DNA docking result obtained by HADDOCK. (DOCX)

S4 Table Number of hydrogen bonds of the native, DNA_contact (R273C and R273H) and rescue mutants (R273C_T284R, R273H_T284R and R273H_S240R) of the p53-DNA complex. (DOCX)
