# Supplementary figures and images for: Structure and Function of p53-DNA Complexes with Inactivation and Rescue Mutations: A Molecular Dynamics Simulation Study
Source: PLoS One. 2015 Aug 5;10(8):e0134638. doi: 10.1371/journal.pone.0134638 (PMC4526489; doi:10.1371/journal.pone.0134638)

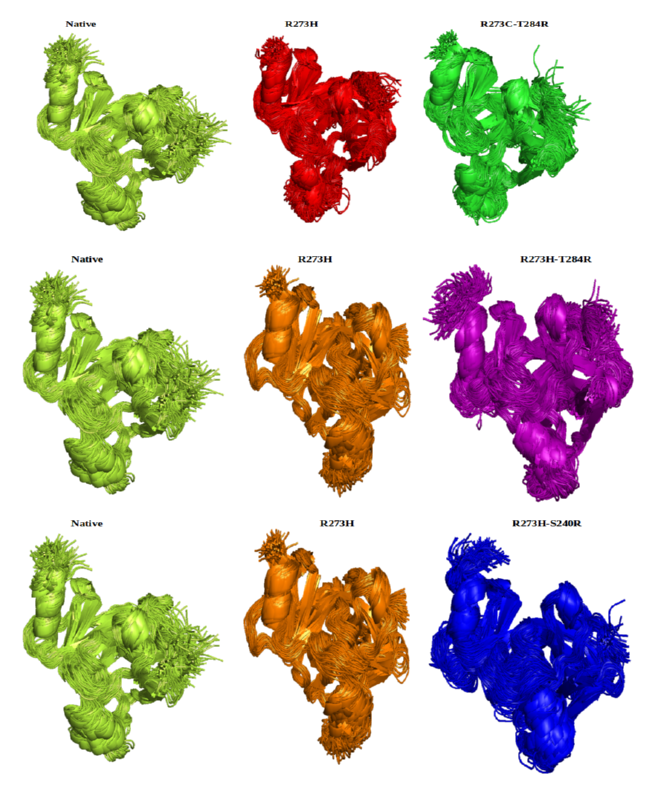

Supplement: S1 Fig — (a) Native, R273C and R273C_T284R, (b) Native, R273H and R273H_T284R, (c) Native, R273H and R273H_S240R. (TIF) [file pone.0134638.s001.tif]
