# Supplementary material for: Structure and Function of p53-DNA Complexes with Inactivation and Rescue Mutations: A Molecular Dynamics Simulation Study
Source: PLoS One. 2015 Aug 5;10(8):e0134638. doi: 10.1371/journal.pone.0134638 (PMC4526489; doi:10.1371/journal.pone.0134638)
Supplement: S1 Table — (DOCX) [file pone.0134638.s002.docx]

**S1 Table:** Average values of RMSD, Rg, SASA and number of hydrogen bonds (NH-bonds) of native p53, DNA-contact (R273C and R273H) and rescue mutants (R273C_T284R, R273H_T284R and R27H_S240R).

| **Parameters** | **Native** | **DNA-contact** | **mutations** | **Rescue** | **mutations** |  |
| --- | --- | --- | --- | --- | --- | --- |
|  |  | **R273C** | **R273H** | **R273C-T284R** | **R273H-T284R** | **R273H-S240R** |
| RMSD (nm) | 0.23 ± 0.03 | 0.17 ± 0.02 | 0.18 ± 0.02 | 0.19 ± 0.03 | 0.23 ± 0.04 | 0.20 ± 0.04 |
| Rg (nm) | 1.68 ± 0.01 | 1.64 ± 0.01 | 1.64 ± 0.01 | 1.66 ± 0.01 | 1.67 ± 0.01 | 1.67 ± 0.01 |
| SASA (nm^2^) | 39.03 ± 1.12 | 37.42 ± 0.96 | 37.7 ± 0.9 | 38.1 ± 0.9 | 38.4 ± 0.9 | 38.3 ± 1.0 |
| NH-bonds | 443.8 ± 11.6 | 455.1 ± 8.7 | 456.2 ± 8.3 | 446.8± 12.5 | 447.4± 12.9 | 445.4 ± 12.5 |
| Covariance value (nm^2^) | 117.03 | 87.36 | 75.37 | 110.69 | 113.68 | 114.46 |
