# Supplementary material for: Structure and Function of p53-DNA Complexes with Inactivation and Rescue Mutations: A Molecular Dynamics Simulation Study
Source: PLoS One. 2015 Aug 5;10(8):e0134638. doi: 10.1371/journal.pone.0134638 (PMC4526489; doi:10.1371/journal.pone.0134638)
Supplement: S2 Table — (DOCX) [file pone.0134638.s003.docx]

**S2 Table:** Results of clustering of the backbone of native, DNA-contact (R273C and R273H) and rescue mutant (R273C_T284R, R273H_T284R and R273H_S240R) structures of the p53 protein structural ensemble obtained from the MD trajectories.

| **Protein Type** | **Total number of clusters** | **Total number of members in the most populated cluster** |
| --- | --- | --- |
| Native | 2589 | 21 |
| R273C | 1699 | 40 |
| R273H | 1581 | 47 |
| R273C_T284R | 2481 | 28 |
| R273H_T284R | 2554 | 25 |
| R273H_S240R | 2392 | 27 |
