# Supplementary material for: Structure and Function of p53-DNA Complexes with Inactivation and Rescue Mutations: A Molecular Dynamics Simulation Study
Source: PLoS One. 2015 Aug 5;10(8):e0134638. doi: 10.1371/journal.pone.0134638 (PMC4526489; doi:10.1371/journal.pone.0134638)
Supplement: S3 Table — (DOCX) [file pone.0134638.s004.docx]

**S3 Table:** Statistical analysis of the protein-DNA docking result obtained by HADDOCK

| **Protein Type** | **HADDOCK score** | **Total interaction energy (Kcal mol-1 )** | **Van der waal**  **energy**  **(Kcal mol-1 )** | **Electrostatic**  **energy**  **(Kcal mol -1 )** | **Desolvation**  **energy**  **(Kcal mol -1 )** | **Restraints**  **violation energy**  **(Kcal mol -1 )** | **Buried surface area**  **A ̊2 )** |
| --- | --- | --- | --- | --- | --- | --- | --- |
| Native | -92.8 ± 2.6 | -156.2 | -81.8 ± 6.7 | -399.0 ± 20.5 | 25.9 ± 3.6 | 431.0 ± 87.0 | 1994.7 ± 87.1 |
| R273C-DNA | -74.2 ± 10.5 | -106.5 | -77.0 ± 4.9 | -342.1 ± 35.8 | 18.1 ± 4.3 | 417.0 ± 64.2 | 1823.3 ± 72.6 |
| R273H-DNA | -73.7 ± 5.9 | -126.9 | -75.2 ± 2.7 | -311.0 ± 30.4 | 18.1 ± 3.8 | 455.6 ± 78.3 | 1845.6 ± 132.1 |
| R273C_T284R-DNA | -85.6 ± 5.7 | -150.8 | -91.8 ± 3.5 | -350.2 ± 19.9 | 29.5 ± 6.3 | 468. 2 ± 40.5 | 2091.2 ± 65.5 |
| R273H_T284R-DNA | -83.9 ± 18.2 | -151.9 | -85.1 ± 9.2 | -420.9 ± 33.6 | 35.1 ± 3.2 | 503.2 ± 109.5 | 2085.8 ± 68.2 |
| R273H_S240R-DNA | -84.3 ± 1.4 | -153.4 | -85.2 ± 8.8 | -384.2 ± 18.3 | 26.1 ± 3.5 | 515.8 ± 72.2 | 1925.7 ± 111.4 |
