# Supplementary material for: Structure and Function of p53-DNA Complexes with Inactivation and Rescue Mutations: A Molecular Dynamics Simulation Study
Source: PLoS One. 2015 Aug 5;10(8):e0134638. doi: 10.1371/journal.pone.0134638 (PMC4526489; doi:10.1371/journal.pone.0134638)
Supplement: S4 Table — (DOCX) [file pone.0134638.s005.docx]

**S4 Table:** Number of hydrogen bonds of the native, DNA_contact (R273C and R273H) and rescue mutants (R273C_T284R, R273H_T284R and R273H_S240R)

of the p53-DNA complex

| **Type of complex** | **Number of H-Bonds** |
| --- | --- |
| Native-DNA | 16 |
| R273C-DNA | 6 |
| R273H-DNA | 6 |
| R273C_T284R-DNA | 16 |
| R273H_T284R-DNA | 18 |
| R273H_S240R-DNA | 21 |
